# CLOCK is an Epigenetic Integrator of Circadian Rhythm and T Cell Immunity

**DOI:** 10.21203/rs.3.rs-9107841/v1

**Published:** 2026-03-27

**Authors:** Shirin Eyvazi, Abu Osman, Abdulrahman Saadalla, Mahendra Pal Singh, Cameron Marlow, Ali Keshavarzian, Faraz Bishehsari, Iradj Sobhani, Aleksey Matveyenko, Fotini Gounari, Khashayarsha Khazaie

**Affiliations:** 1 Department of Immunology, Mayo Clinic, Phoenix, Arizona, United States, USA; 2 Department of Pathology, University of Utah School of Medicine, Salt Lake City, Utah, USA; 3 Department of Internal Medicine RUSH, Chicago, Illinois, USA; 4 Department of Internal Medicine, University of Texas Health Science Center, Houston, Texas, USA; 5 Department of Physiology and Biomedical Engineering, Mayo Clinic, Rochester, Minnesota, USA; 5 Department of Gastroenterology Hospital Henri Mondor, APHP, Creteil, France.

**Keywords:** Epigenetics, CLOCK, BMAL1, Circadian Rhythm, Immune Signaling, Naïve CD4 T Cell Lineage Specification

## Abstract

The circadian clock imposes a critical yet incompletely understood layer of regulation on adaptive immunity. T helper 17 (Th17) antimicrobial immune responses including expression of IL-17A by lamina propria CD4^+^ T cells, exhibit diurnal variation and are sensitive to circadian disruption. While the core circadian regulators CLOCK and BMAL1 canonically function as a heterodimer to drive rhythmic gene expression, emerging evidence suggests they may have distinct regulatory roles. Here we integrated ChIP-seq, ATAC-seq, and RNA-seq analyses in naïve CD4^+^ T cells to reveal that the CLOCK-BMAL1 complex controls circadian and metabolic programs through promoter binding, whereas exclusive CLOCK binding at promoters, together with CLOCK-BMAL1 binding at enhancers, regulates immune-associated genes. Using the mutant CLOCK^Δ19^, which lacks the transactivation domain, we observed disrupted circadian transcription and globally reduced chromatin accessibility, alongside increased accessibility at Th17-associated loci and altered their temporal regulation. Functionally, CLOCK^Δ19^ produces β-catenin stabilization in T cells, pronounced expansion of RORγt^+^ CD4^+^ T cells and Treg cells, impaired Treg suppression of Th17 responses, heightened Th17 responses, and reduced IFN-γ production during viral infection. Collectively, these findings define BMAL1 dependent and independent CLOCK functions that program naïve CD4^+^ T cell fate, restrain Th17 differentiation, and preserve immune homeostasis.

## Introduction

The immune system is under tightly circadian control, with both innate and adaptive responses exhibiting time of day dependent oscillations. These rhythms are driven by intracellular transcriptional-translational feedback loops composed of circadian clock genes that regulate their own expression and that of cellular function in a time dependent manner. Among the core regulators of the circadian clock, the transcription factors CLOCK and BMAL1 (ARNTL) form a heterodimeric complex that binds E-box elements in DNA to regulate rhythmic gene transcription. This regulatory system influences a wide array of physiological processes, including development, cell trafficking, metabolism, as well as cancer ^1, 2, 3^, and is increasingly recognized as a critical layer of immune regulation ^4, 5, 6^. CD4^+^ T cells possess intrinsic circadian oscillators, contributing to diurnal variations in cytokine production and surface molecule expression ^7, 8, 9^. Rhythmic expression of key migratory receptors such as CCR7 and S1PR1 on lymphocytes enables their oscillatory trafficking through lymph nodes, leading to daily variations in lymphocyte cellularity and antigen responsiveness ^10, 11^. In parallel, the circadian regulation of antigen-presenting cells, particularly dendritic cells, influences the strength of T cell priming, with BMAL1 regulating mitochondrial dynamics and calcium homeostasis to modulate antigen processing capacity in a circadian manner ^12^. The clinical relevance of these circadian immune dynamics is increasingly evident in gut health ^13^ and in determining the therapeutic efficacy and adverse-event profiles of vaccines and cancer therapies ^14^. Together, these findings indicate that circadian timing mechanisms are integral to T cell biology, shaping the timing, quality, and magnitude of adaptive immune responses. However, the precise mechanisms by which core clock genes regulate T cell immunity remain incompletely understood.

Recent studies demonstrates that immune regulation of gut microbiota is under circadian control ^15^ and compromise of diurnal rhythms increase the risk of colitis ^16^ and intestinal carcinogenesis ^17, 18^. Notably, the circadian transcriptional repressor REV-ERBα and NFIL3 have emerged as key modulators of Th17 inflammation, regulating expression of REV-ERBα and RORγt to influence T cell lineage specification and function^7, 19^. Additionally, BMAL1 deficiency has been associated with impaired development and functionality of type 3 innate lymphoid cells (ILC3s) ^20, 21, 22, 23^. In contrast, a study using T cell-specific BMAL1 knockout mice suggest that BMAL1 is dispensible for T cell development and function ^24^, leaving the contribution of circadian genes to Th17 cell differentiation and function uncertain.

There is strong evidence that circadian rhythms regulate gene expression through epigenetic modifications and changes in chromatin structure^25, 26^. Importantly, these circadian effects are highly tissue specific, with limited overlap in rhythmic gene expression across different tissues^27^. Consistent with this, the binding of the core clock proteins BMAL1^28^ and CLOCK^29^ to DNA regulatory elements is also largely tissue specific. Together, these findings indicate that core clock genes establish tissue- and developmental stage-specific epigenetic and transcriptional programs. However, it remains unclear how these circadian regulatory mechanisms influence the differentiation of naïve CD4^+^ T cells into the Th17 lineage^28^. Understanding these mechanisms will clarify how circadian regulation governs Th17 lineage commitment, functional specialization, and inflammatory potential.

To directly investigate the role of the core circadian regulators, CLOCK and BMAL1, in the functional specification of T cells, we used *Clock*^Δ19^ mutant mice ^30^. This mutation deletes exon 19, producing a CLOCK protein that can still heterodimerize with BMAL1 and bind DNA, but has reduced transcriptional activity at target regulatory elements, resulting in the disruption of feedback loops that generate the 24-hour cycle ^30^. *Clock*^*Δ19*^ mice on a C57BL/6 background display an extended circadian period, with a free-running period of ~28 hours in constant darkness compared to the ~24 hours in wild type mice. They also show progressively reduced circadian rhythmicity, leading to arrhythmia and phase-shifted expression of clock regulated genes ^31^. We combined flow cytometry-based phenotyping and functional differentiation assays, with transcriptional and chromatin profiling. Specifically we performed bulk RNA-seq, ATAC-seq and mapped the genome wide binding of CLOCK and BMAL1 using ChIP-seq to define the molecular mechanisms by which CLOCK regulates CD4^+^ T cell differentiation. To assess cell-intrinsic effects of both CLOCK and the CLOCK^Δ19^ mutant while minimizing confounding effects from activation or differentiation, we focused on naïve CD4^+^ T cells which are transcriptionally quiescent and functionally inactive. We performed analysis on cells isolated from mesenteric lymph nodes (MLN), as it is an immune compartment that is responsive to microbial and environmental cues.

We demonstrate that CLOCK exerts distinct functions together with or without BMAL1. Through heterodimerization with BMAL1 it regulates circadian rhythmicity, while independently of BMAL1 it modulates immune programs. These activities are mediated by selective interactions with promoter and enhancer elements across the genome. Notably, the observed epigenetic alterations occur in naïve T cells in the absence of overt Th17 or Treg effector transcription, indicating that CLOCK establishes lineage competence through epigenetic priming. Collectively, our findings identify CLOCK as a central regulator of inflammatory responses by directing T helper cell polarization and modulating Treg function.

## Results

### 1. Circadian Disruption Drives Uncontrolled Th17 Inflammation.

Earlier studies have demonstrated that disruption of the light-dark cycle increases intestinal Th17 cell frequencies and heightens susceptibility to inflammatory disease ^7^. These studies suggest that Th17 cell development is regulated by the circadian clock network in part through direct regulation of *Rorc* (encoding RORγt). Building upon this, we examined the temporal regulation of IL-17A production by intestinal CD4^+^ T cells at two key circadian time points in wild-type (WT) mice: ZT23 (just before the onset of the rodent inactive phase; 5am) and ZT11 (just prior the onset of active phase, 5pm).

**Figure 1.**
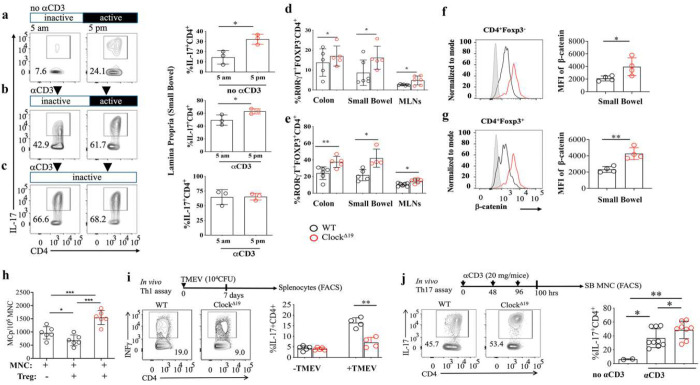
The circadian CLOCK regulates intestinal Th17 cell frequencies and effector function. (a–c) 4 months old wild-type (WT) were analyzed for Th17 responses under homeostatic conditions and following intraperitoneal injection of anti-CD3 antibody. Representative flow cytometry plots (left) and cumulative bar graphs (right) show IL-17A^+^ CD4^+^ T cell frequencies in the small intestinal lamina propria at circadian time points ZT23 and ZT11 (n = 3 per group; *P < 0.05). Mice were maintained either under standard 12:12 light-dark (LD) cycles with right-time feeding (RF) (a-b) or constant light (LL) conditions (c). (d&e) Frequencies of RORγT^+^FOXP3^−^ conventional CD4^+^ T cells (Tcon) and RORγt^+^FOXP3^+^ regulatory T cells (Treg cells) in WT and *Clock*^*Δ19*^ mice were assessed in the mesenteric lymph nodes (MLNs), small intestine, and colon (n = 5–7 per group; MLNs: *P < 0.04, **P < 0.006). (f&g) Intracellular β-catenin expression levels in Tcon and Treg cells were determined by flow cytometry; histograms (left) are normalized to mode, and cumulative mean fluorescence intensity (MFI) data are shown (right) (n = 4; *P < 0.02). (h) Suppression of mast cell progenitors by FOXP3^+^ Treg cells from WT and Clock^Δ19^ mice in an in vitro co-culture assay (***P < 0.0004). (i) Quantification of Th1 responses following Theiler’s murine encephalomyelitis virus (TMEV) infection. On day 7 post-infection, IFN-γ expression in spleen-derived CD4^+^ T cells was measured by intracellular flow cytometry (n = 5; *P < 0.001). (j) Th17 responses were further quantified in WT and Clock^Δ19^ mice after anti-CD3 challenge (n = 5; *P < 0.01, **P < 0.003). All graphs show mean – s.e.m. from biologically independent animals. Data are representative of at least two independent experiments. Statistical significance was assessed using two-sided unpaired t-tests.

Four months old mice were randomly assigned to two entrainment conditions: (1) a standard 12:12 LD cycle and (2) constant light (LL; >100 lux, 25-watt fluorescent bulbs placed 12 inches above the cages). After 10 weeks of entrainment, mice were sacrificed at ZT11 or ZT23. Under steady-state conditions in the 12:12 LD, flow cytometric analysis revealed significantly higher IL-17A expression in lamina propria CD4^+^ T cells at ZT11 compared to ZT23 (*P* < 0.04; Figure 1ab). To assess whether this oscilating IL-17A expression is dependent on intact circadian cues, we maintained a separate cohort of WT mice under constant light (LL) for 10 weeks. This environmental circadian disruption abolished the diurnal oscillation of IL-17A expression in gut-resident CD4^+^ T cells by sustaining high levels of IL-17 at ZT23 (Figure 1c). These findings are consistent with the circadian regulation of IL-17 production by CD4^+^ T cells, and chronic Th17 function.

To assess the role of CLOCK and CLOCK transactivation domain in Th17 lineage specification, we compared WT mice with mice expressing a dominant negative *Clock*^*Δ19*^ lacking transactivation function^30^. Analysis of the mutant mice at ZT4 (10 am; beginning of the inactive period) revealed a significant increase in the frequency of RORγT^+^FOXP3^−^ conventional CD4^+^ T cells (*P* < 0.04; Figure 1d) and RORγT^+^FOXP3^+^ regulatory T (Treg) cells (*P* < 0.005; Figure 1e) in the colon, small intestine, and MLNs. These changes were accompanied by a significant increase in β-catenin stabilization in both CD4^+^ T cells (*P* < 0.04; Figure 1f) and Treg cells (*P* < 0.0004; Figure 1g) isolated from the small intestine of *Clock*^*Δ19*^ mice, suggesting that *Clock*^*Δ19*^ stabilizes β-catenin in CD4^+^ T cells and Treg cells. Cell intrinsic β-catenin / TCF-1 signaling in T cells and Treg cells has previously been linked by our group to activation, expansion, and gain of proinflammatory properties by Th17 cells and RORγT^+^ Treg cells, in colitis, polyposis, and CRC ^32, 33^. Building on these findings, we compared WT and *Clock*^Δ19^ Treg cells and found that the *Clock*^Δ19^ mutation markedly diminishes the ability of Treg cells to suppress mast cell maturation (*P* < 0.03; Figure 1h), consistent with gain of proinflammatory properties ^34^. Together, these findings indicate that loss of CLOCK transactivation enhances β-catenin signaling and drives a proinflammatory shift in CD4^+^ T cells and Treg cells, highlighting a key role for CLOCK in restraining Wnt/β-catenin–dependent T cell programs.To further substantiate the type of Treg suppressive functions that were compromised, we assessed regulation of Th1 and Th17 responses in WT and *Clock*^Δ19^ mice. For Th1 polarization, mice were infected with *Theiler’s murine encephalomyelitis virus* (TMEV), and mononuclear cells were isolated from the spleen on day 7 post-infection. Cells were stimulated *ex vivo* with phorbol 12-myristate 13-acetate (PMA) and ionomycin in the presence of GolgiStop to assess intracellular IFN-γ production*.* CD4^+^ T cells in *Clock*^Δ19^ mice expressed significantly less IFN-γ in response to virual challenge than in WT controls (P < 0.0002; Figure 1i), indicating markedly dampened Th1 response. To evaluate Th17 responses, we utilized a previously established anti-CD3 antibody challenge protocol^15^. Four days post-injection, CD4^+^ T cells from the small-intestinal lamina propria were analyzed for IL-17A expression by flow cytometry. *Clock*^Δ19^ CD4^+^ T cells showed higher IL-17A expression compared to WT counterparts (P < 0.04, P < 0.003; Figure 1h), indicating exaggerated Th17 response. Collectively, these findings demonstrate that within the gut environment, CLOCK regulates Th17 and Th1 responses in part by altering properties of Treg cells. Loss of the CLOCK transactivation domain leads to expansion of Th17 cells while selectively impairing Treg-mediated suppression of the cells. In contrast CLOCK transactivation domain appears to be needed for effective Th1 response, since it’s loss dampens anti-viral Th1 response.

### 2. CLOCK^Δ19^ Rewires Circadian, Epigenetic, RNA-Regulatory and Stress-Response Programs in Naïve CD4^+^ T Cells.

**Figure 2.**
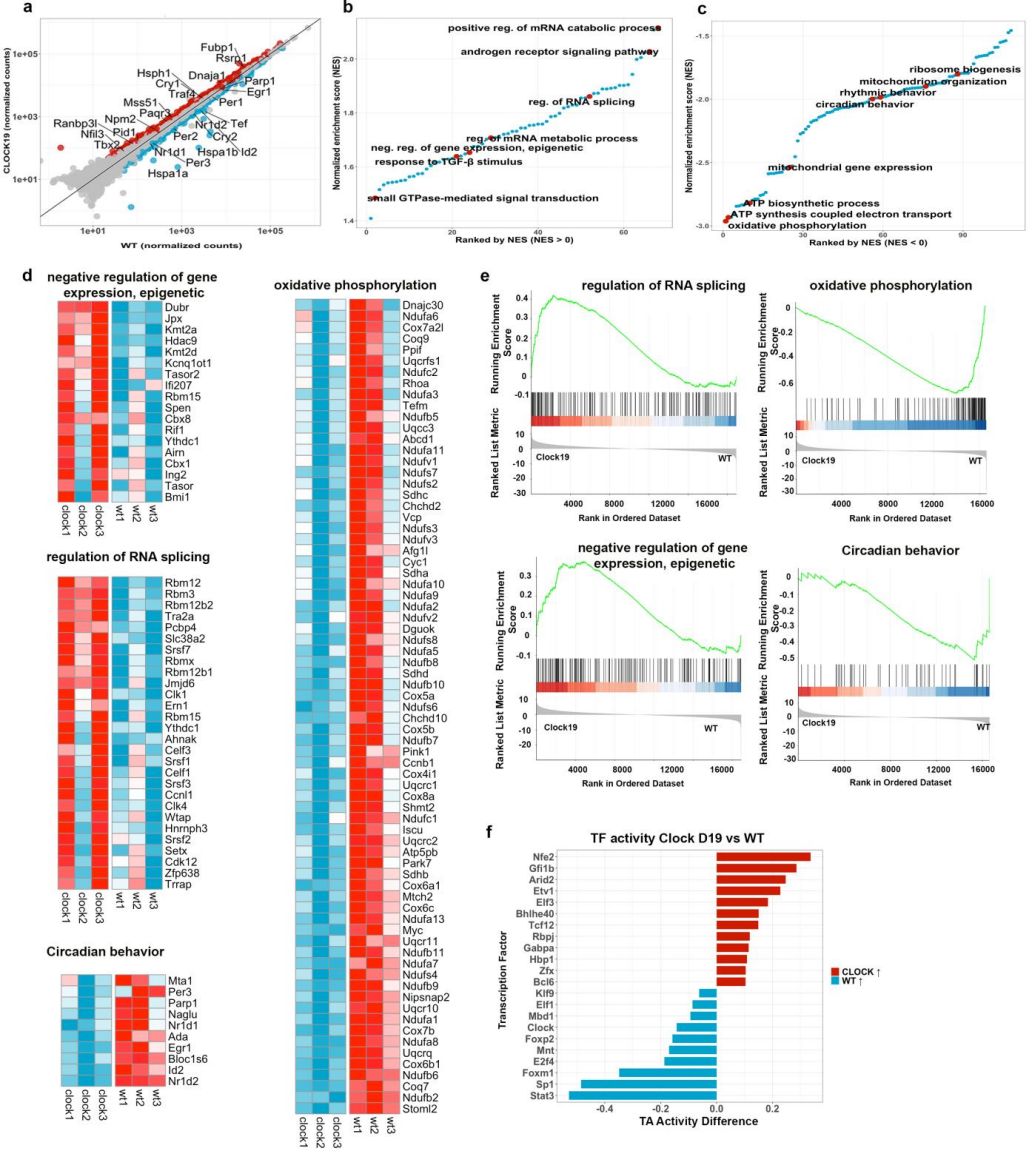
*Clock*^Δ19^ Rewires Circadian, Epigenetic, RNA-Regulatory and Stress-Response Programs in Naïve CD4^+^ T Cells. **(a)** Scatterplot showing normalized gene expression levels in naïve CD4^+^ T cells from *Clock*^Δ19^ versus WT mice. Core circadian regulators (e.g., *Per2, Per3, Tef, Nr1d1/REV-ERBα, Nr1d2/REV-ERBβ*) and stress related proteins (*Hspa1a, Hspa1b,Traf4*) are among the most strongly deregulated transcripts in *Clock*^Δ19^ cells. **(b–c)** Ranked normalized enrichment scores (NES) from GSEA highlight pathways upregulated (b) or downregulated (c) in *Clock*^Δ19^ naïve T cells. **(d)** Heatmaps showing normalized expression of leading-edge genes from the selected enriched GO terms, comparing *Clock*^Δ19^ and WT samples. **(e)** Corresponding GSEA running-enrichment plots illustrating enrichment direction and leading-edge gene contributions for selected pathways. **(f)** VIPER-based transcription factor activity analysis shows increased inferred activity of lineage-priming and stress-response factors in naïve *Clock*^Δ19^CD4^+^ T cells.

To better understand the immune alterations observed in *Clock*^Δ19^ mice, we analyzed the transcriptional profiles of naïve CD4^+^ T cells early in the inactive period at ZT4, early in the inactive period when immune activity and Th17 responses is reduced. *Clock*^Δ19^ naïve T cells showed significant downregulation of several core circadian regulators, including *Per2, Per3, Tef, Nr1d1 (*REV-ERBα*)*, and *Nr1d2 (*REV-ERBβ*)* (Figure 2a). Expression of *Nfil3* and *Cry1,* which are normally repressed by REV-ERBα, were elevated. These findings were corroborated by gene set enrichment analysis (GSEA), which shows overall down regulation of circadian behavior (Figure 2 c,d,e, **Supplementary Table 1**) but enhanced epigenetic regulation of gene expression (*Cbx8, Bmi1*, Polycomb-associated regulator) (Figure 2 b,d,e).

The *Clock*^*Δ19*^ mice experienced altered expression of stress-associated genes compared to WT mice. Here we observed the upregulation of the chaperon proteins *Hsph1*, *Dnaja-1* and HSP4, golgi protein *Paqr3*, and reduced expression of the stress inducible heat shock proteins *Hspa1* and *Hspa1b,* suggesting changes in protein-quality control (Figure 2a). Furthermore, RNA metabolism appeared as a prominent target of *Clock*^Δ19^, with differential expression of genes involved. These included mechanisms of RNA splicing, stability and metabolism (*Rbm12, Rbm3, Pcbp4, Wtap, Ern1*) (Figure 2b,d,e, **Supplementary Table 2**), aligning with the role of circadian regulators in the rhythmic control of RNA metabolism^35^. The androgen signaling pathway which modulates Treg functions under chronic inflammatory conditions^36^ and the TGF-β stimulus responses that critically contribute to the generation of extrathymic Rorγt^+^ Treg cells, were highly upregulated in *Clock*^Δ19^ naïve T cells, suggesting a major impact on Treg differentiation and suppressive functions (Figure 2b). Using Virtual Inference of Protein-activity by Enriched Regulon analysis (VIPER), we identified transcription factor programs associated with chromatin remodeling, stress responses, and early T cell lineage priming in naïve *Clock*^*Δ19*^ CD4^+^ T cells. This was reflected by increased inferred activity of factors including *Nfe2, Arid2, Elf3, Etv1, Tcf12, Bhlhe40, and Gfi1b*). In contrast, WT T cells showed higher inferred activity of transcription factors associated with transcriptional restraint and metabolic homeostasis *(e.g., Mnt, E2f4, Sp1, and Foxp2)* as well as greater CLOCK activity, consistent with disruption of core circadian regulatory programs in *Clock*^Δ19^ cells (Figure 2 f). Together, these findings identify CLOCK as a key regulator of naïve CD4^+^ T cell lineage programming, coordinating circadian, epigenetic, RNA-regulatory, and stress-response pathways that shape T cell maturation and functional potential.

### 3. CLOCK^Δ19^ Alters Chromatin Accessibility and Circadian Dynamics in Naïve CD4^+^ T Cells.

**Figure 3.**
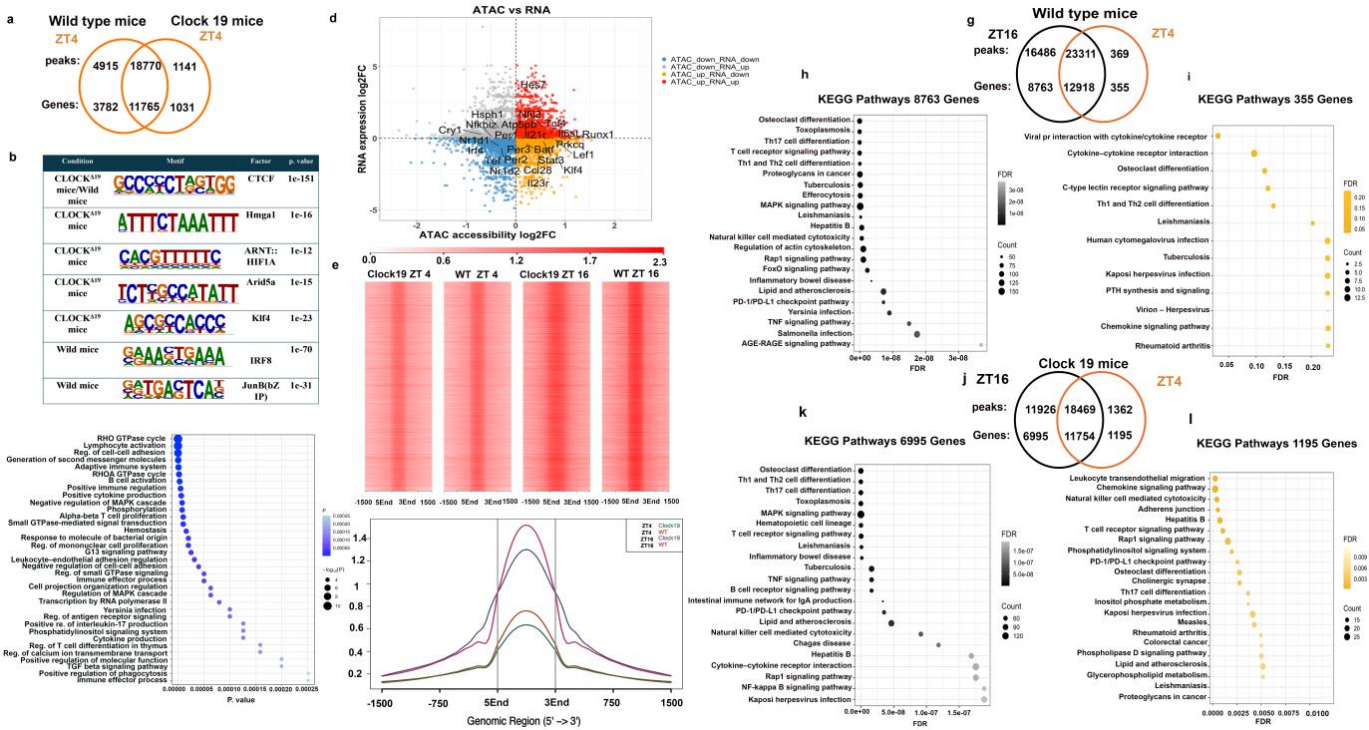
CLOCK^Δ19^ Disrupts Both Chromatin Accessibility and Its Circadian Dynamics in Naïve CD4 T Cells. **(a)** Venn diagram showing differential chromatin accessibilities and overlaps between WT and Clock^Δ19^ naïve CD4 T cells based on ATAC-seq peaks and associated genes. **(b)** DNA motifs enriched in regions uniquely accessible in WT or *Clock*^Δ19^ cells. **(c)** Increased accessibility of genes associated with immune activation in *Clock*^Δ19^ cells based on Metascape pathway enrichment analysis. **(d)** Increased accessibility of Th17 associated genes in the absence of transcription in Clock^Δ19^ cells, assessed by integrated ATAC-seq and RNA-seq analysis. **(e)** Heatmaps of chromatin accessibility around transcription start sites (TSS) (±1.5 kb) for naïve CD4 T cells isolated from wild-type and *Clock*^Δ19^ mice at morning (ZT 4) and night (ZT 16). Each column represents aggregated ATAC-seq signal across all genes. **(f)** Average signal profiles of accessible chromatin regions centered on the TSS (±1.5 kb) in different conditions. **(g)** Venn diagram showing overlap between ZT 4 and ZT16 accessible peaks and their associated target genes in WT mice. **(h-i)** KEGG pathway enrichment for specific genes associated with specific accessible peaks in Wild Type mice. **(j)** Venn diagram showing overlap between ZT 4 and ZT16 accessible peaks and their associated target genes in *Clock*^Δ19^. (k-l) KEGG pathway enrichment for specific genes associated with specific accessible peaks in *Clock*^Δ19^ mice.

To determine how the *Clock*^Δ19^ mutation alters chromatin accessibility, we performed ATAC-seq on naïve CD4 T cells from WT and *Clock*^Δ19^ mice in early resting phase at ZT4. *Clock*^Δ19^ cells showed a global reduction in accessible chromatin compared to WT cells (1,141 vs 4,915), although a substantial number of accessible sites (18,770) were shared between mutant and WT cells (Figure 3a). *De novo* motif enrichment analysis performed using HOMER ^37^ revealed that regions uniquely accessible in both genotypes were enriched for CTCF motifs, consistent with higher order chromatin organization, in contrast CLOCK^Δ19^-specific regions were enriched for HMGA1 motifs, suggesting reduced chromatin openness (Figure 3b). Examination of genes associated with HMGA1-enriched regions, as well as CTCF-associated regions, identified multiple epigenetic regulators linked to chromatin compaction and repressive programs. These included, Kdm2b, which connects to Polycomb Repressive Complex 1 (PRC1)^38^, and Sumo1, implicating SUMO-dependent chromatin modification^39^. In addition, chromatin-associated factors such as Asf1a (a histone chaperone involved in nucleosome assembly^40^) and Hmgb2 (a chromatin structural protein) were detected within these motif-enriched regions. Together, these findings suggest that some accessible chromatin in *Clock*^Δ19^ CD4^+^ cells may be poised for compaction and recruitment of Polycomb- and heterochromatin-associated complexes.

Additionally, CLOCK^Δ19^-specific accessible chromatin regions were enriched for the conserved motifs of the transcription factors Arid5a and Irf1 (Figure 3b), both previously implicated in Th17 cell differentiation. Gene annotation of CLOCK^Δ19^-associated accessible regions (1,030 genes) revealed significant enrichment for pathways related to T cell differentiation and Th17-associated functions, including IL-17 production (Figure 3c). These motif and pathway enrichment analyses align with our flow cytometry and *in vitro* polarization data, demonstrating enhanced Th17 differentiation in *Clock*^Δ19^ cells (Figure 1). In contrast, our analysis of genes associated with WT-specific accessible regions (3,782 genes) showed enrichment for broader naïve CD4 T-cell differentiation programs encompassing Th1, Th2, and Th17 lineages, as well as T-cell receptor signaling pathways (Supplementary Figure 1, **Supplementary Table 3**).

Integration of ATAC-seq and RNA-seq datasets identified coordinated changes in chromatin accessibility and gene expression that were specific to *Clock*^Δ19^ cells (Figure 3d). Several Th17-associated loci including *Il23r, Batf, Stat3,* and *Klf4*, showed increased chromatin accessibility in the absence of corresponding changes in transcript levels, indicating epigenetic priming. However, core circadian genes (*Per1–3, Cry1, Tef, Nr1d1,* and *Nr1d2*) displayed reduced accessibility and decreased expression, consistent with disruption of the circadian transcriptional program. Collectively, these chromatin accessibility profiles together with our *in vitro* functional data support a model in which naïve CD4 T cells in *Clock*^Δ19^ mice are epigenetically poised toward proinflammatory Th17 and Rorγt^+^ Treg differentiation trajectories. Importantly, this reprogramming is not accompanied by overt transcriptional activation of Th17 effector genes, thereby consistent with the maintenance of a phenotypically naïve state prior to lineage commitment.

Having established that the *Clock*^Δ19^ mutation reduces overall chromatin accessibility in naïve CD4 T cells, we next investigated whether this mutation also disrupts the circadian regulation of chromatin accessibility. To examine diurnal changes in chromatin accessibility we performed ATAC-seq at ZT16 (active phase) in naïve CD4^+^ T cells from WT and *Clock*^*Δ19*^ mice and compared it with the ATAC-seq profiling from ZT4 (resting phase),. (Figure 3 e–f). In WT cells, chromatin accessibility was markedly increased at ZT16 compared with ZT4 (16,486 vs. 369 peaks; 8,763 vs. 355 genes). *Clock*^Δ19^ cells also showed higher accessibility at ZT16 than at ZT4 (11,926 vs. 1,362 peaks; 6,995 vs. 1,195 genes), however, the magnitude of this increase was significantly attenuated relative to WT (Figure 3-g&j, **Supplementary Table 3**).

Pathway enrichment analysis of time point-specific accessible regions revealed that in WT naïve CD4 T cells, Th17 differentiation pathways were enriched only at ZT16, together with Th1/Th2 differentiation, TCR signaling, MAPK signaling, and infection-related immune pathways (Figure 3h). This pattern is consistent with previous reports showing that Th17 responses peak during the nocturnal active phase in mice. In comparison, WT cells at ZT4 showed no enrichment for Th17 differentiation and instead displayed a more restricted profile dominated by cytokine-cytokine receptor interactions and chemokine signaling pathways (Figure 3i). The findings in WT cells were remarkably constrasted in *Clock*^*Δ19*^ cells. In these cells, ZT4 and ZT16 exhibited strong enrichment of Th17 differentiation, cytokine/chemokine signaling, and TCR signaling, with additional Th1/Th2 pathways emerging at ZT16 (Figure 3k–l). These findings indicate that loss of the CLOCK transactivation domain enhances and sustains Th17-associated chromatin accessibility during the resting phase, which is normaly characterized by reduced inflammatory pntential.

### 4. CLOCK and CLOCK-BMAL1 Differentially Regulate Immunity and Circadian Control.

**Figure 4.**
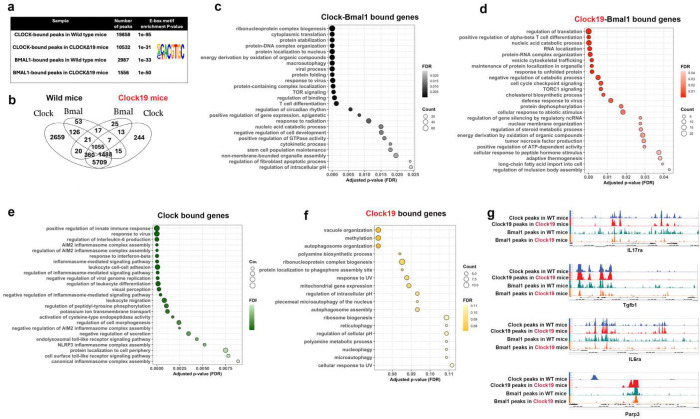
CLOCK and CLOCK-BMAL1 Differentially Regulate Immunity and Circadian Control. (a) Enriched DNA-binding motifs identified by ChIP-seq for CLOCK and BMAL1 in WT and *Clock*^Δ19^ mice.(b) Venn diagram showing regulatory elements co-bound or uniquely bound by CLOCK and BMAL1 in WT mice, or by CLOCK^Δ19^ and BMAL1 in mutant mice. (c–f) Pathway enrichment analyses of genes co-bound by CLOCK–BMAL1 in WT mice (c), CLOCK^Δ19^–BMAL1 in mutant mice (d), exclusively bound by CLOCK in WT mice (e), or exclusively bound by CLOCK^Δ19^ in mutant mice (f). (g) ChIP-seq peak profiles at representative Th17-associated gene loci bound by CLOCK or CLOCK^Δ19^.

We next used ChIP seq analysis to examine the genome-wide binding patterns of CLOCK and BMAL1 in naïve CD4^+^ T cells. WT and *Clock*^*Δ19*^ mice were analysed early in the inactive period at ZT4, when immune activity and Th17 responses normally decline. These analyses revealed both shared and distinct chromatin binding patterns for CLOCK and BMAL1. CLOCK binding was markedly reduced in *Clock*^Δ19^ cells compared to WT cells, with 10,532 peaks and 19,858 peaks respectively. Similarly, BMAL1 binding was decreased in *Clock*^Δ19^ cells, with 2,987 peaks in WT cells versus 1,556 peaks in *Clock*^Δ19^ cells (Figure 4a). Peak annotation using HOMER linked CLOCK- and BMAL1-bound regions to putative target genes based on genomic proximity. In both genotypes, substantially more genes were associated with CLOCK binding alone than co-occupied by the CLOCK-BMAL1 heterodimer. In WT cells, CLOCK bound regions mapped to 8,829 genes, whereas CLOCK^Δ19^ bound regions mapped to 7,537 genes. By comparison, regions co-occupied by CLOCK-BMAL1 were associated with 2,690 genes in WT and 1,435 genes in *Clock*^Δ19^ cells (Figure 4b). Importantly, CLOCK^Δ19^ failed to bind regions associated with 2,659 loci that were normally targeted by WT CLOCK and instead gained binding at 259 novel loci. Similarly, regions associated with the CLOCK^Δ19^-BMAL1 heterodimer lost binding at 1,635 WT CLOCK-BMAL1 associated loci and instead mapped to 380 novel genes (Figure 4b, **Supplementary Table 4**).

Functional annotation of genes linked to these gain- and loss-of-binding events revealed distinct regulatory programs. Genes associated with WT CLOCK-BMAL1 binding were enriched for circadian and rhythmic processes as well as immune pathways including T cell differentiation (Figure 4c). Importantly, CLOCK-BMAL1 binding was enriched at genes involved in epigenetic regulation of gene expression, suggesting a direct role in epigenetic control, consistent with patterns observed in the RNA-seq and ATAC-seq datasets. When we examined the genes bound by the CLOCK^Δ19^-BMAL1 heterodimer, we observed enrichment for stress-response pathways, including DNA, RNA, and protein metabolism, cell-cycle checkpoints, protein localization to organelles, unfolded protein response pathways, and T cell differentiation (Figure 4d).

Functional annotation of these gain- and loss-of-binding events revealed distinct regulatory programs. Genes associated with WT CLOCK-BMAL1 binding were enriched for circadian and rhythmic processes and immune pathways including T cell differentiation (Figure 4c). Notably, CLOCK-BMAL1 binding enriched for pathways involved in epigenetic regulation of gene expression, consistent with pattenrs observed in RNA-seq and ATAC-seq datasets. In contrast, genes uniquely associated with CLOCK^Δ19^-BMAL1 binding were enriched for stress-response pathways, including DNA, RNA, and protein metabolism, cell-cycle checkpoints, protein localization to organelles, unfolded protein response pathways, as well as T cell differentiation (Figure 4d).

**Figure 5.**
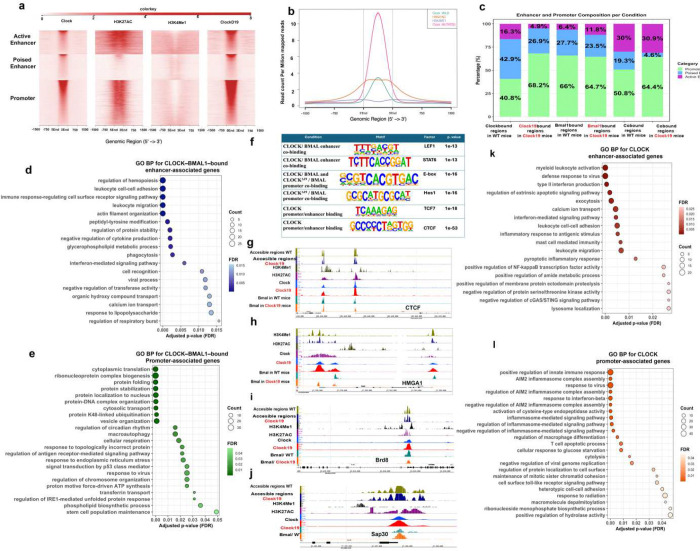
Promoter and Enhancer Binding by CLOCK and CLOCK-BMAL1 Differentially Regulate Immunity and Circadian Control. (a) Heatmaps of CLOCK ChIP-seq signal intensity centered on CLOCK-bound regions in WT and Clock^Δ19^ mutant mice. (b) Average ChIP-seq signal profiles for CLOCK and CLOCK^Δ19^, H3K27ac, and H3K4me1 centered on CLOCK peak regions. (c) Stacked bar plot showing distributions of CLOCK-only and CLOCK^Δ19^ -only bound or with BMAL1co-bound regions across promoters, poised enhancers, and active enhancers in WT and mutant samples. (d) Gene ontology biological process enrichment analysis of genes associated with CLOCK-BMAL1 co-binding at enhancers, (e) and promoters. (f) Enriched DNA motifs identified under distinct CLOCK and BMAL1 binding conditions across regulatory elements. (g-j) IGV tracks showing CLOCK, BMAL, and histone-mark ChIP-seq peaks as well as ATAC peaks at representative gene loci. (k)Gene ontology biological process enrichment analysis of genes associated with CLOCK binding at enhancers, (l) and promoters. Bars represent the top enriched biological processes ranked by −log_10_ (p-value).

Exclusive CLOCK binding in WT cells was largely associated with immune-related genes, with enrichment for type 1 and type 3 immune pathways, including interferon-β signaling, antiviral response, AIM2 inflammasome activation, interleukin-6 signaling, pattern recognition receptor pathways, and positive regulation of immune responses (Figure 5e). We did not detect CLOCK^Δ19^ bound regions at these same immune loci and instead instead, binding was redistributed to a smaller set of genes, including *Dnmt3b* and *Rnmt*, which are involved in DNA and RNA methylation (Figure 4f). We used Integrated Genome Browser (IGB) software for comparative analysis of ChIP-seq peaks from both genotypes and observed differential binding across multiple regulatory elements at immune-related loci (Figure 4g), providing a framework for subsequent analysis of these regulatory regions.

### 5. Promoter and Enhancer Binding by CLOCK and CLOCK-BMAL1 Differentially Regulate Immunity and Circadian Control.

E-box (bHLH) motifs are enriched in both promoter and enhancer regions. To define CLOCK and BMAL1 occupancy at these regulatory elements, we clustered binding sites based on enrichement of histone marks. Publicly available ChIP-seq datasets for histone marks in naïve CD4 T cells were obtained from GEO and aligned to the mus musculus (mm) 10 genome and analysed using NGSplot assisted clustering to identify regulatory regions (Figure 5a, b, c). Enhancers were defined by enrichement of H3K4me1 and classified as active when co-marked by H3K27ac, whereas active promoters were identified by H3K27ac enrichment in the absence of H3K4me1. CLOCK and BMAL1 ChIP-seq peaks were assigned to these regulatory categories (Figure 5a). Despite having fewer overall binding sites (Figure 5a,b), CLOCK^Δ19^ exhibited increased average ChIP-seq signal intensity at its bound regions, suggesting a more selective chromatin-binding profile (Figure 5b).

Compared with CLOCK, CLOCK^Δ19^ showed preferential binding at promoter regions (68.2%) and reduced occupancy at poised (26.9%) and active enhancers (4.9%) (Figure 5c). The CLOCK-BMAL1 heterodimer showed greater enhancer engagement than CLOCK alone, with increased occupancy at active enhancers (30%) and reduced binding at poised enhancers (19.3%), while maintaining substantial promoter binding (50.8%). CLOCK^Δ19^-BMAL1 and WT CLOCK-BMAL1 heterodimers displayed similar distributions at promoter (50.8% vs 64.4%) and active enhancers (30% vs 30.9%) respectively. These patterns were independently validated using HOMER-based annotation relative to transcription start sites (Supplementary Figure 2), indicating that BMAL1 is required for efficient CLOCK recruitment to enhancers, consistent with its role in enhancer organization and chromatin architecture ^28, 41^.

Functionally, CLOCK-BMAL1 binding at enhancers was associated with immune-related genes, including *Runx1, Cd28, Il6st, Cxcr5*, and *Nfatc3* (Figure 5d), whereas promoter binding preferentially marked circadian regulators (*Usf1, Bmal1, Rora, Cipc*) and genes involved in DNA/RNA metabolism, protein synthesis, and cell-cycle control (Figure 5e, Supplementary Figure 3). In contrast, CLOCK^Δ19^-BMAL1 heterodimer chromatin occupancy at enhancers associated with ER stress-responsive genes such as *Ddit3*, a central regulator of the unfolded protein response (UPR) (Supplementary Figure 4a). Similarly, CLOCK^Δ19^-BMAL1 promoter binding was enriched for genes linked to ER stress, DNA repair, cell cycle check point, catabolic processes, and post-transcriptional or post-translational modifications (Supplementary Figure 4b). Together, these data indicate that while CLOCK-BMAL1 heterodimer primarily regulates immunity and circadian rhythmicity via enhancer and promoter engagement respectivley, the CLOCK^Δ19^ mutation perturbs this division of labor, redirecting both promoter and enhancer-associated binding towards stress-related transcriptional programs.

Our examination and analysis of binding motifs further revealed the role of chromatin architecture in circadian rhythmicity. CLOCK-BMAL1 co-bound enhancers were enriched for immune-associated transcription factor motifs, including LEF1 and STAT6, whereas promoters associated regions showed strong enrichment for circadian motifs (E-box, ARNTL, USF1) together with factors linked to chromatin conformation, metabolism, proliferation, and cell cycle control (CTCF, TFE3, MYB) (Figure 5f). In the CLOCK^Δ19^-BMAL1 context, promoter motifs included Hes1, accompanied by increased Hes7 expression in our RNA-seq data, suggesting altered Notch signaling, a pathway known to interface with circadian regulators ^42^. Notably, enhancer-promoter-associated targets included CTCF, and promoter-associated targets included HMGA1, both of which were identified as differentially accessible motifs in ATAC-seq analyses, indicating direct regulation of chromatin architectural factors by CLOCK-BMAL1. This regulatory axis extended to epigenetic co-factors, including Brd8 and Sap30 (Figure 5g–j). Brd8 was transcriptionally upregulated in our RNA-seq dataset and showed direct CLOCK and BMAL1 occupancy at its regulatory regions. Similarly, Sap30 was overexpressed and displayed increased chromatin accessibility enriched for HMGA-associated motifs. Collectively, these findings suggest that CLOCK-BMAL1 engages chromatin architectural and epigenetic regulators, linking transcription factor binding to coordinated changes in chromatin accessibility and gene expression programs in naïve CD4 cells.

We also observed that exclusive CLOCK occupancy at enhancers versus promoters distinguished distinct transcriptional programs. For instance, enhancer bound regions by CLOCK or CLOCK^Δ19^ were enriched for immune-related pathways, including leukocyte activation and inflammatory responses (Figure 5k&l; Supplementary Figure 4c), whereas promoter CLOCK^Δ19^ binding was preferentially associated with metabolic, stress response and inflammatory pathways (Supplementary Figure 4d). Our motif analysis supported these findings by revealing enrichment of LEF1, TCF7 and CTCF motifs at CLOCK-bound sites, but not at CLOCK^Δ19^-bound sites (Figure 5f).

Collectively, these findings suggest that immune-related gene regulation is preferentially associated with CLOCK binding at promoters and CLOCK-BMAL1 binding at enhancers, whereas circadian regulation is primarily mediated by CLOCK-BMAL1 promoter occupancy.

To determine how chromatin binding relates to transcriptional output, we integrated ChIP-seq and RNA-seq datasets. Cumulative distribution function (CDF) analysis showed that genes uniquely bound by CLOCK^Δ19^ displayed a left-shifted log_2_ fold-change distribution compared with genes bound by CLOCK or CLOCK–BMAL1, consistent with transcriptional repression, although this difference did not reach statistical significance (p = 0.74; Supplementary 5a).

Integration of ATAC-seq with ChIP-seq further revealed that CLOCK- and BMAL1-bound regions were less accessible than the global chromatin landscape, with a further reduction in accessibility in Clock^Δ19^ cells (Supplementary 5b). CDF analysis confirmed a significant shift toward lower accessibility at CLOCK^Δ19^ co-bound regions (Kolmogorov–Smirnov test, p < 0.05; Supplementary 5c), supporting increased chromatin compaction. Notably, binding by CLOCK or CLOCK^Δ19^, alone or with BMAL1, was associated with a greater reduction in chromatin accessibility at promoters than at enhancers (Supplementary 5d), indicating that promoter occupancy preferentially contributes to chromatin closing.

Together, these integrative analyses support a model in which loss of CLOCK transactivation enhances promoter-associated chromatin compaction and attenuates gene expression.

## Discussion

Circadian regulation is increasingly recognized as a fundamental determinant of immune function, yet how intrinsic clock machinery shapes adaptive immune fate decisions remained unclear. In this study, we defined a central role for the circadian transcription factor CLOCK in programming naïve CD4 T cells through epigenetic and transcriptional mechanisms that precede lineage commitment. By focusing on naïve T cells from mesenteric lymph nodes, we highlighted primary cell-intrinsic effects of disrupting CLOCK functions within a gut-associated immune environment that is highly sensitive to microbial and dietary cues. The use of *Clock*^Δ19^ mice allowed us to focus on CLOCK intrinsic functions associated with its transactivation domain.

We uncovered a division of labor between CLOCK and CLOCK-BMAL1 complexes and their binding to promoters versus enhancers. Our ChIP-seq analysis showed that CLOCK-BMAL1 primarily enforces diurnal oscillation and metabolic homeostasis through promoter-centered regulation, consistent with its canonical role in circadian transcription. We observed that CLOCK alone binds to promoters whereas CLOCK-BMAL1 bind to enhancers that regulate immune-associated genes. This demonstrate both division of labor and functional overlap to coordinate immune responses with circadian rhythmicity.

Notably, the temporal ATAC-seq analyses showed that CLOCK normally restricts Th17-associated chromatin accessibility to specific circadian phases, aligning immune readiness with time-of-day cues. Loss of CLOCK transactivation function abolished this rhythmic behavior, resulting in sustained accessibility of Th17 regulatory regions throughout the circadian cycle. This loss of temporal restriction contributes to exaggerated and persistent Th17 response. Chromatin accessibility profiling using ATAC-seq revealed that *Clock*^Δ19^ naïve CD4 T cells adopt a globally more compact chromatin state, yet exhibit increased accessibility at Th17-associated regulatory elements. Transcriptomic profiling of naïve CD4^+^ T cells showed disruption of core circadian, metabolic, and stress-response pathways, rather than direct induction of Th17 effector genes. This suggests that the loss of CLOCK transactivation domain alters the basal cellular state in a way that primes cells for Th17 differentiation before lineage commitment. Together, these findings highlight a key role for CLOCK in guiding naïve T cell lineage specification and establishing antimicrobial defense programs before full lineage commitment occurs.

Our findings suggest that circadian control of gene expression depends not only on rhythmic transcription factor binding but also on dynamic remodeling of chromatin architecture ^43^. Aguilar-Arnal *et al.* ^44^ demonstrated that CLOCK-BMAL1 target genes undergo rhythmic three-dimensional reorganization, including enhancer-promoter looping and nuclear repositioning, that coincides with transcriptional activation in the liver. Similarly, Yuan *et al.* ^45^ showed that circadian rhythms of chromatin accessibility are cell-type specific and regulated by core clock components, with rhythmic opening and closing of cis-regulatory elements. These findings underscore the importance of time-of-day-dependent chromatin remodeling in circadian transcription. CTCF and HMGA motifs were among the most enriched elements for *Clock*^Δ19^ binding, indicating a fundamental role of CLOCK in regulating higher order chromatin structure, a function that has been largely attributed to BMAL^28^. Together, these findings identify CLOCK as an epigenetic gatekeeper that programs chromatin accessibility and circadian timing in naïve CD4 T cells. CLOCK does not simply regulate immune gene expression but establishes temporal and epigenetic constraints that limit when and how differentiation programs can be engaged.

The circadian clock represents a critical yet incompletely defined regulator of adaptive immunity. By integrating intestinal T cell responses *in vivo*, we showed that IL-17A production by lamina propria CD4^+^ T cells follow a diurnal pattern and that disruption of the CLOCK transactivation domain in *Clock*^Δ19^ mice results in pronounced immune dysregulation. This included expansion of RORγt^+^ CD4^+^ T cell subsets, β-catenin stabilization, impaired Treg suppressive function, exaggerated Th17 responses, and diminished IFN-γ production during viral infection. The genetic impairment of CLOCK transactivation functions leads to altered CD4^+^ T cell lineage balance, thereby promoting differentiation of CD4 T cells into RORγt-expressing Th17 and RORγt^+^ Treg populations.

Notably, *Clock*^Δ19^ RORγt^+^ Treg cells display elevated β-catenin levels and reduced suppressive capacity toward mast cells, indicating impaired regulatory functions. This phenotype resembles the expanded RORγt^+^ Treg cells popuplaton observed in patients with CRC and IBD and in corresponding mouse models ^34, 46, 47, 48^. Furthermore, our earlier studies demonstrated that β-catenin stabilization or TCF-1 ablation produce similar immune outcomes ^33, 49, 50^, supporting a functional link between CLOCK and canonical Wnt signaling. Our findings highlighted an important role of CLOCK dysfunction in immune dysregulation in cancer and chronic inflammatory diseases. Notably, *Clock*^Δ19^ RORγt^+^ CD4^+^ T cells and Treg cells displayed elevated β-catenin levels and pro-inflammatory properties, indicating selectively impaired mucosal immune regulation. The *Clock*^Δ19^ Treg phenotype resembles those of the earlier described RORγt^+^ Treg cells that expand in patients with CRC or IBD and in corresponding mouse models ^34, 46, 47, 48^. Furthermore, our earlier studies demonstrate that, β-catenin stabilization or TCF-1 ablation produce similar changes in the properties of T cells and Treg cells ^33, 49, 50^, supporting a functional link between CLOCK and canonical Wnt signalling

Our combined findings indicate that the CLOCK transactivation domain functionally interfaces with chromatin modifiers and the Wnt/β-catenin pathway in naïve CD4^+^ T cells to regulate lineage specification and effector function. Loss of this domain skews early CD4^+^ T cell competence toward pathogenic Th17 polarization at the expense of Th1 responses causing immune imbalances that naturally occur in cancer and chronic inflammatory diseases. These findings establish a mechanistic basis for CLOCK-dependent regulation of T helper cell and Treg differentiation and function. In this context, CLOCK emerges as a central integrator of circadian and immune signaling in naïve CD4^+^ T cells.

### Impact Statement

This study establishes CLOCK as a cell-intrinsic epigenetic gatekeeper that links circadian timing to adaptive immune fate decisions. By delineating BMAL1-dependent and independent functions of CLOCK in naïve CD4^+^ T cells, we uncover a division of labor that coordinates immune responses with diurnal gene expression. Our findings further suggest that the CLOCK transactivation domain interfaces with chromatin remodeling and canincal Wnt signaling to determine CD4 T cell lineage specification prior to T cell differentiation. These findings provide a mechanistic framework connecting circadian misalignment to deregulated host responses to environmental disrupters and Th17-driven pathologies.

## Material and methods:

### Methods

#### Animals and Experimental Design

This study employed homozygous *Clock*^Δ19^ mice, which harbor a dominant-negative deletion of exon 19 in the core circadian transcription factor Clock, resulting in disrupted circadian regulatory function. These animals, originally generated on a C57BL/6J genetic background, are widely recognized for their disrupted circadian function, impacting rhythmicity at the molecular, physiological, and behavioral levels ^30, 51, 52, 53^. Wild-type (WT) littermates (C57BL/6J ) were used as controls to ensure genetic consistency. This mutant model is well-characterized and extensively employed in circadian biology research to investigate links between rhythmic dysregulation and broader physiological outcomes^54, 55, 56^.

All procedures were approved by the Institutional Animal Care and Use Committee (IACUC) at Mayo Clinic and performed under specific pathogen-free (SPF) housing conditions. Mice were maintained under standard environmental parameters, including a controlled ambient temperature (22–23°C), 60% humidity, and a 12-hour light/12-hour dark cycle. Food and water were available ad libitum throughout the study. Unless otherwise indicated, both male and female mice aged 4–5 months were used, and all animals were euthanized at Zeitgeber time 4 (ZT4). For circadian light entrainment experiments, 10-week-old mice were randomly allocated to two groups. One group remained under the standard 12:12 light-dark (LD) cycle with feeding aligned to their active period (right-time feeding, RF). The second group was exposed to constant light (LL) conditions at an intensity exceeding 100 lux using 25-watt fluorescent lights placed 12 inches above the cages, also paired with RF. This regimen continued for 10 weeks, after which animals were sacrificed at two circadian time points: ZT11 (just before the onset of active phase) and ZT23 (just before the onset of rest phase).

### Tissue Processing and Cell Isolation

#### MLNs and Spleen:

Tissues were mechanically dissociated through 40-μm cell strainers (Falcon). Red blood cells in splenocyte suspensions were lysed with ACK buffer (Lonza) for 1 minute on ice, followed by washing in PBS containing 2% FBS (F8067; Sigma).

#### Intestine (Small Bowel and Colon):

After removing fat and longitudinally opening the tissue, samples were minced and enzymatically digested in a solution containing collagenase IV (12 mg, LS004188; Worthington), DNase (180 U, D5025; Sigma), and hyaluronidase (1.2 mg, H3506; Sigma) in 20 ml RPMI 1640 with constant stirring at 37 °C for 25 minutes. Digestion was performed twice. Resulting cell suspensions were filtered and washed in PBS-2% FBS. Mononuclear cells were enriched by centrifugation over a Percoll gradient (44% over 67%, *P1644;* Sigma) at 2400 × rpm for 18 minutes at 4 °C without braking. The interface was collected and washed prior to analysis.

### Flow Cytometry

Single-cell suspensions were stained with LIVE/DEAD Fixable Blue Stain (Invitrogen, L34962; 1:750 dilution) to exclude non-viable cells, followed by incubation with fluorochrome-conjugated surface antibodies for 30 minutes at 4 °C. The following antibodies were used for surface staining: CD4 (clone RM4–5; BioLegend), conjugated to either PerCP/Cy5.5 or Brilliant Violet 785 (1:300); CD25-BV560 (clone PC61; BioLegend; 1:200); and CD8a-V500 (clone 53–6.7; BD Biosciences; 1:200).

For detection of transcription factors, surface-stained cells were fixed and permeabilized using the FOXP3/Transcription Factor Staining Buffer Set (eBioscience, 00–5523-00). Intracellular staining was performed with the following antibodies: FOXP3 (clone FJK-16s; FITC or APC; eBioscience, 1:200), RORγt (clone Q31–378; BV421 or PE; BD Biosciences, 1:200), and β-catenin-Alexa Floor FITC (clone 14/β-Catenin; BD Biosciences, 5 μL/sample). Staining was carried out for overnight at 4 °C, followed by two washes with permeabilization buffer.

For phospho-protein analysis, lymphocytes were incubated in complete medium for 1 hour at 37 °C, fixed with BD Phosflow Lyse/Fix buffer (BD, 558049), and permeabilized using Phosflow Perm Buffer III (BD, 558050). Cells were then stained with FITC-conjugated anti–phospho-STAT3 (Tyr705; clone 11–9033-42; eBioscience, 5 μL/test) or FITC-conjugated Mouse IgG2b κ isotype control (eBioscience; 1 μg/test).

Samples were acquired using a BD LSR Fortessa X-20 flow cytometer and analyzed with FlowJo software (Tree Star).

### Viral Infections

For induction of acute viral infection, mice were infected intraperitoneally on day 0 with 2.5–5.0 × 10^3^ plaque-forming units of TMEV (kindly provided by Dr. Kevin Pavelko, Mayo Clinic) suspended in plain DMEM.

### *In vivo* TH1 Polarization and IFN-γ Staining

WT and *Clock*^Δ19^ mice were intraperitoneally (*i.p.*) injected with Theiler’s murine encephalomyelitis virus (TMEV) and euthanized 7 days post-infection. Spleens were harvested and processed into single-cell suspensions. Cells were stimulated with 50 ng/ml phorbol-12-myristate-13-acetate (PMA; Sigma, P1585) and 0.75 μg/ml ionomycin (Sigma, 13909) for 5 hours in the presence of 1 μg/ml GolgiStop (BD Biosciences, 555029). Surface staining was followed by intracellular staining for IFN-γ (clone XMG1.2, eBioscience, 17–7311-82; dilution 1:200).

### *In vivo* TH17 Polarization and IL-17A Staining

Mice received i.p. injections of anti-CD3 antibody (20 μg per mouse; clone 2C11, BioLegend) or PBS at 0, 48, and 96 hours, following established protocols^57^. At 100 hours post-initial injection, small intestines were harvested. Intraepithelial and lamina propria lymphocytes were isolated by enzymatic digestion and restimulated with PMA–ionomycin for 5 hours. Intracellular IL-17A staining was performed (clone TC11–18H10, BD Biosciences, 559502; dilution 1:300).

### RNA Isolation and RNA Sequencing

Approximately ~ 3× 10^4^ naïve CD4^+^ T cells were isolated from the mesenteric lymph nodes and spleens of wild-type (WT) and *Clock*^Δ19/Δ19^ mutant mice using the Mouse Naive CD4^+^ T Cell Isolation Kit (Miltenyi Biotec), following the manufacturer’s protocol. Total RNA was extracted using the PicoPure RNA Isolation Kit (Arcturus) according to the manufacturer’s instructions. RNA integrity and quantity were assessed prior to library preparation. RNA libraries were prepared and sequencined at the Translational Genomics Research Institute (TGen) Genomics Facility (Phoenix, AZ).

### Chromatin Immunoprecipitation and Sequencing (ChIP–seq)

Approximately 1 × 107 naïve CD4^+^ T cells were isolated from mesenteric lymph nodes and spleens of WT and ClockΔ19/Δ19 mice at ZT4 using the Mouse Naïve CD4^+^ T Cell Isolation Kit (Miltenyi Biotech, USA), according to the manufacturer’s instructions. Cells were crosslinked in suspension with 1% formaldehyde at room temperature for 10–15 min with gentle agitation, and crosslinking was quenched by addition of glycine to a final concentration of 125 mM. Cells were washed twice with ice-cold PBS and lysed in hypotonic lysis buffer (10 mM Tris-HCl pH 8.0, 10 mM NaCl, 0.2% Igepal CA-630) supplemented with EDTA-free protease inhibitors to isolate nuclei.

Nuclei were resuspended in SDS-containing buffer and incubated briefly at elevated temperature to solubilize chromatin, followed by dilution in shearing buffer. Chromatin was fragmented by focused ultrasonication using a Covaris ME220 with the following parameters: duty cycle 5%, cycles per burst 200, total sonication time 10 min, to obtain DNA fragments predominantly in the 200–500 bp range, as verified by agarose gel electrophoresis or TapeStation analysis. Sheared chromatin was clarified by centrifugation, and an aliquot was reserved as input control.

Immunoprecipitation was performed by incubating clarified chromatin overnight at 4°C with antibodies against BMAL1 (Cell Signaling Technology) or CLOCK (Abcam) in the presence of blocking reagents, including fish gelatin (2%) and heparin as indicated. Immune complexes were captured using Protein A/G magnetic beads (Thermo Fisher Scientific) and washed sequentially to reduce nonspecific binding with high-salt RIPA buffer (10 mM Tris-HCl pH 7.4, 1 mM EDTA, 1% Triton X-100, 0.1% sodium deoxycholate, 0.1% SDS, 0.5 M NaCl), followed by a wash with LiCl buffer (0.25 M LiCl, 0.5% NP-40, 0.5% sodium deoxycholate), and a final wash with TE buffer.

Chromatin was eluted from beads in SDS-containing elution buffer, and crosslinks were reversed by prolonged incubation at 65°C in the presence of NaCl. Samples were treated sequentially with RNase A and proteinase K (Ambion, Thermo Fisher Scientific). ChIP DNA was purified by phenol–chloroform extraction followed by ethanol precipitation and resuspended in elution buffer.

Purified ChIP DNA was processed for sequencing library preparation using the NEBNext Ultra II DNA Library Prep Kit for Illumina, including end repair, A-tailing, adaptor ligation, and cleanup using AMPure XP beads. Library amplification was performed using indexed primers, with the optimal number of PCR cycles determined by qPCR to minimize amplification bias. Enrichment was assessed by quantitative PCR using primers targeting the Per1 promoter as a positive control and an unrelated genomic region as a negative control *. Final libraries were evaluated for fragment size distribution and concentration prior to pooling and sequenced on an Illumina NovaSeq X Plus platform at the TGen Genomics Facility (Phoenix, AZ).

### Assay for Transposase-Accessible Chromatin Using Sequencing (ATAC-seq)

Naïve CD4^+^ T cells (~50,000 per sample) were purified from the mesenteric lymph nodes and spleens of *Clock*^Δ19/Δ19^ and WT mice using the Mouse Naïve CD4^+^ T Cell Isolation Kit (Miltenyi Biotec), in accordance with the supplier’s protocol. Following isolation, cells were centrifuged at 500 × g for 5 minutes at 4 °C, washed in PBS, and pelleted again under the same conditions. To lyse the cells and isolate nuclei, the pellet was gently resuspended in cold lysis buffer (10 mM Tris-HCl, pH 7.4; 10 mM NaCl; 3 mM MgCl_2_; 0.1% IGEPAL CA-630) and immediately centrifuged at 500 × g for 10 minutes at 4 °C. Nuclei were then subjected to the transposition reaction using a mix containing 25 μl of 2× Tagment DNA buffer, 2.5 μl of Tagment DNA enzyme (Illumina, FC-121–1030), and 22.5 μl of nuclease-free water. The reaction was incubated at 37 °C for 30 minutes. Tagmented DNA was purified using the Qiagen MinElute Reaction Cleanup Kit and subsequently amplified by PCR using Illumina Nextera Index primers and NEBNext PCR Master Mix (New England BioLabs, M0541). Final libraries were cleaned using a Qiagen PCR purification kit and sequenced on the Illumina HiSeq 4000 platform at the University of Chicago Genomics Facility^49^.

### RNA-seq data processing, differential expression, and transcription factor activity analysis

Raw RNA-seq reads were subjected to adapter and quality trimming using Trimmomatic (v0.39; https://github.com/usadellab/Trimmomatic). Trimmed reads were aligned to the mouse reference genome using the STAR aligner (v2.7; https://github.com/alexdobin/STAR), allowing for accurate spliced read alignment. Only uniquely mapped reads were retained for downstream analyses. Gene-level read counts were generated from aligned BAM files using featureCounts from the Subread package (v2.0; http://subread.sourceforge.net/) based on annotated gene models.

Count matrices were imported into R (v4.2 or later; https://www.r-project.org/) for downstream statistical analyses. Differential gene expression analysis was performed using DESeq2 (https://bioconductor.org/packages/DESeq2), with normalization and dispersion estimation conducted according to the standard DESeq2 framework. Differential expression results were ranked by log_2_ fold change and used for pathway-level analyses.

To infer transcription factor (TF) activity from RNA-seq data, we applied a VIPER-based approach (Virtual Inference of Protein-activity by Enriched Regulon analysis), which estimates regulator activity based on the coordinated expression of target gene regulons rather than TF expression levels alone. TF activity inference was performed using the VIPERalgorithm implemented in R (https://bioconductor.org/packages/viper), leveraging curated and inferred regulatory networks. Differential TF activity scores were calculated between experimental conditions and used to identify key transcriptional regulators associated with observed transcriptional and epigenomic changes.

### Pathway and functional enrichment analysis

Gene set enrichment analysis (GSEA) was performed using preranked gene lists to identify significantly enriched biological pathways (https://www.gsea-msigdb.org). Functional enrichment analyses were conducted using curated pathway databases, including Gene Ontology Biological Process (GO BP) (https://geneontology.org) and KEGGpathways (https://www.kegg.jp). Integrated enrichment and pathway clustering analyses were additionally performed using Metascape (https://metascape.org). Multiple testing correction was applied where appropriate.

### ChIP-seq and ATAC-seq data processing and analysis

ChIP-seq and ATAC-seq data processing was performed using the Galaxy web-based analysis platform (https://usegalaxy.org) to ensure reproducibility and standardized workflows. Raw sequencing reads from ChIP /ATAC samples, as well as FASTQ files for histone modification datasets downloaded from the NCBI Gene Expression Omnibus (GEO) (https://www.ncbi.nlm.nih.gov/geo/), were subjected to adapter trimming and quality filtering using Trimmomatic (v0.39; https://github.com/usadellab/Trimmomatic).

Trimmed reads were aligned to the mm10 mouse reference genome using Bowtie (http://bowtie-bio.sourceforge.net), retaining only uniquely mapped reads. Aligned BAM files were filtered to remove low-quality reads, and PCR duplicates were identified and removed using Galaxy-implemented tools to minimize amplification bias prior to peak calling. Peak detection was performed using MACS2 (Model-based Analysis of ChIP-Seq; https://github.com/macs3-project/MACS) with matched input controls and the mm10 genome build. To generate a unified and non-redundant peak set across samples and conditions, individual peak files were merged using the HOMER mergePeaks function (http://homer.ucsd.edu/homer/). Peak annotation and assignment to nearby genes were carried out using HOMER, which was also used for transcription factor motif enrichment analysis within peak regions.

Visualization of ChIP-seq and ATAC-seq signal intensity across genomic features, including promoters and transcription start sites, was performed using ngs.plot (https://github.com/shenlab-sinai/ngsplot), generating average enrichment profiles and heatmaps. Additional data processing, visualization, and statistical analyses were conducted in R(https://www.r-project.org/).

### ATAC-seq differential accessibility analysis

ATAC-seq peaks corresponding to accessible chromatin regions were quantified across samples, and differential chromatin accessibility between experimental conditions was assessed using standard statistical frameworks implemented in R. Differentially accessible regions were defined based on fold-change and adjusted P-value thresholds. Accessible regions were annotated using HOMER and classified based on genomic context and overlap with regulatory elements.

Genes associated with differentially accessible regions were subjected to functional enrichment analysis using curated pathway databases, including Gene Ontology Biological Process (GO BP) (https://geneontology.org) and KEGG pathways (https://www.kegg.jp). Integrated pathway enrichment and clustering analyses were additionally performed using Metascape (https://metascape.org).

## Supplementary Material

Supplement 1

Supplementary Files

This is a list of supplementary files associated with this preprint. Click to download.

• SupplementaryTable1.downpathwaystranscriptionClock19vswild.csv

• SupplementaryTable2.uppathwaystranscriptionClock19vswild.csv

• SupplementaryTable4.geneslistforchipseq.csv

• SupplementaryTable3.geneslistforATACSeq.csv

## Data Availability

The sequencing data generated in this study have been deposited in the Gene Expression Omnibus (GEO) under accession number GSE325743. Raw sequencing data have been submitted to the NCBI Sequence Read Archive (SRA) under the associated BioProject accession PRJNA1425474. The data will be made publicly available upon publication. During peer review, the data are available from the corresponding author upon reasonable request.
