# Supplementary material for: CLOCK is an Epigenetic Integrator of Circadian Rhythm and T Cell Immunity
Source: Res Sq. 2026 Mar 27:rs.3.rs-9107841. Preprint. [Version 1] doi: 10.21203/rs.3.rs-9107841/v1 (PMC13042162; doi:10.21203/rs.3.rs-9107841/v1)
Supplement: Supplement 1 [file NIHPPrs9107841v1-supplement-1.pdf]

Supplimentary Figures:

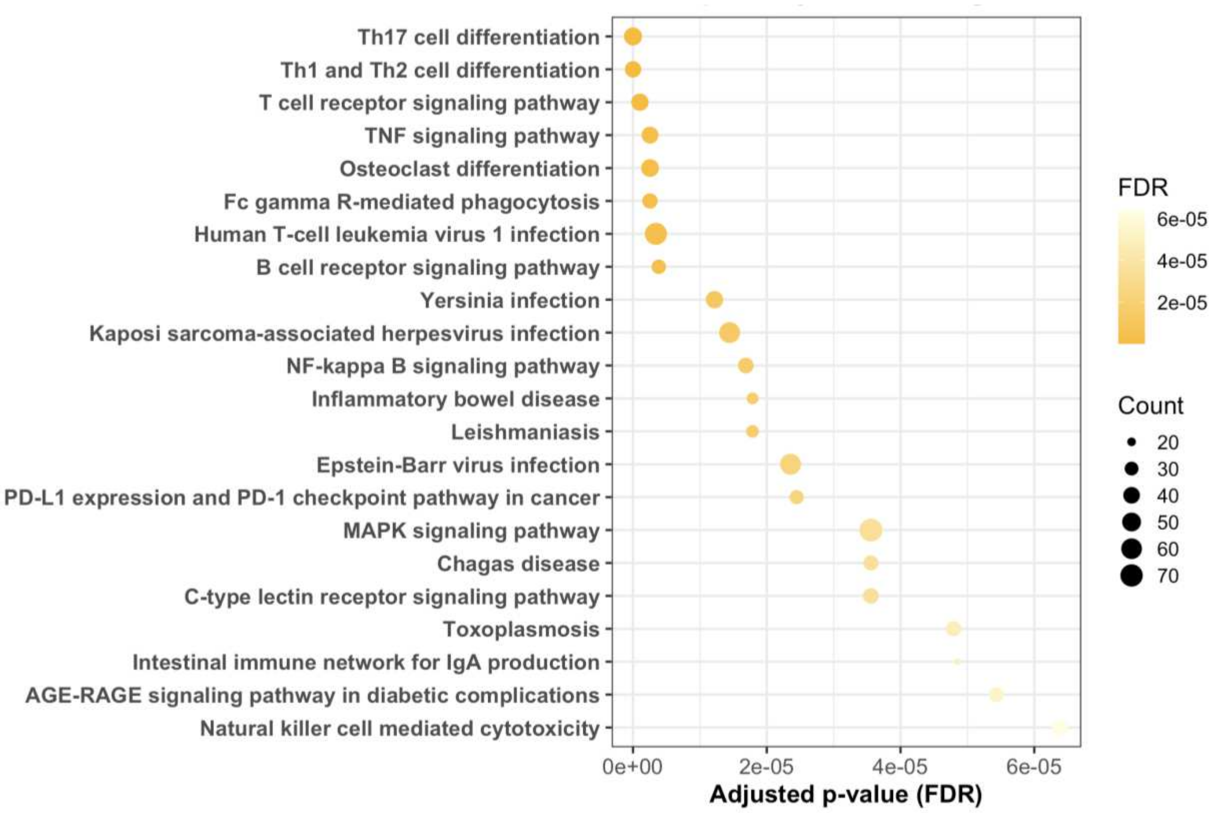

Supplimentary_Figure 1. GO and KEGG pathway enrichment analysis of genes with increased chromatin accessibility in WT during the morning (ZT 4) compared with ClockΔ19. Dot size represents the number of genes (Count) associated with each GO term, and color intensity indicates the FDR**.**

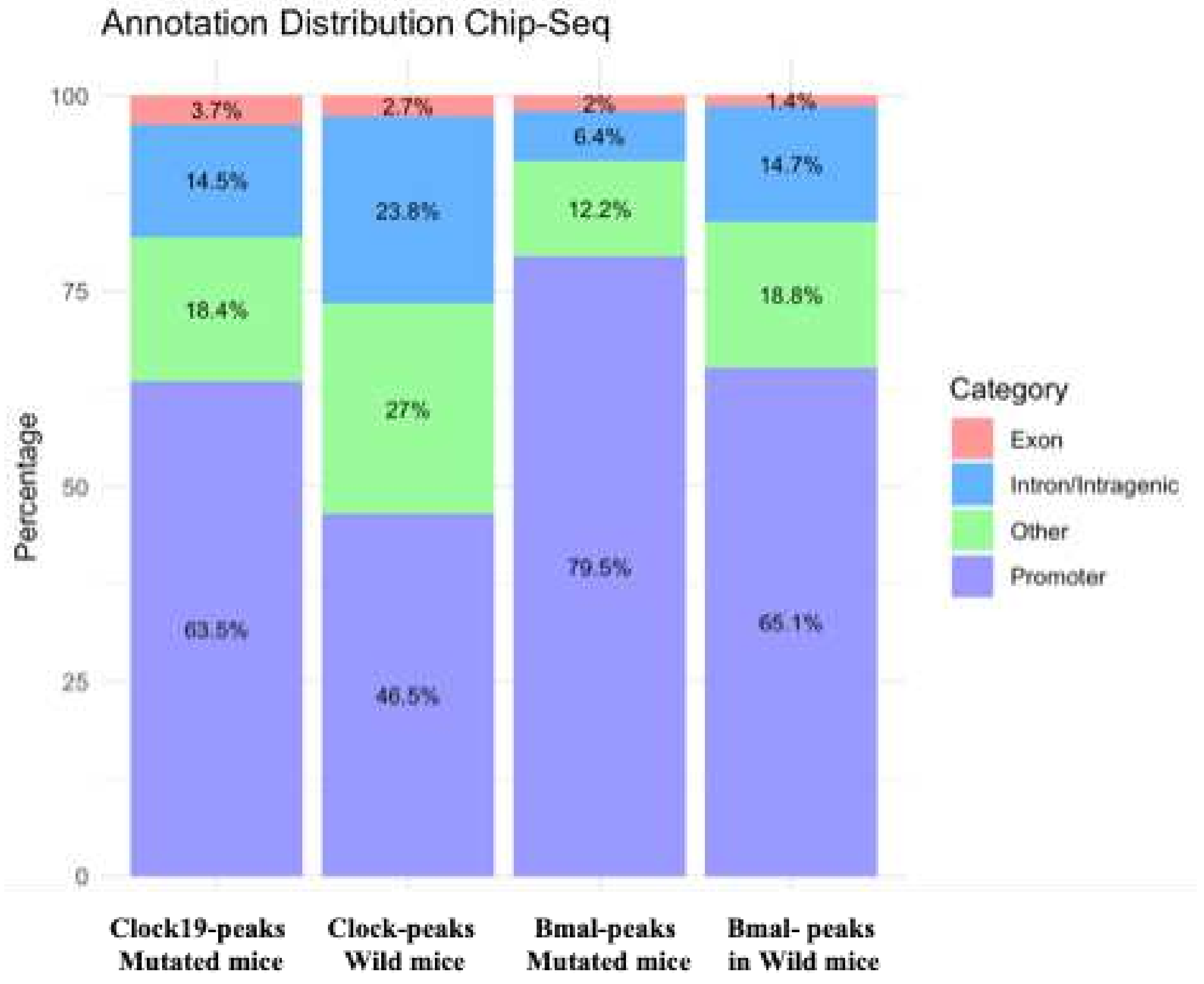

Supplimentary_Figure 2 Anotation of Chip-Seq peaks relative to transcription start sites (TSS) using HOMER.

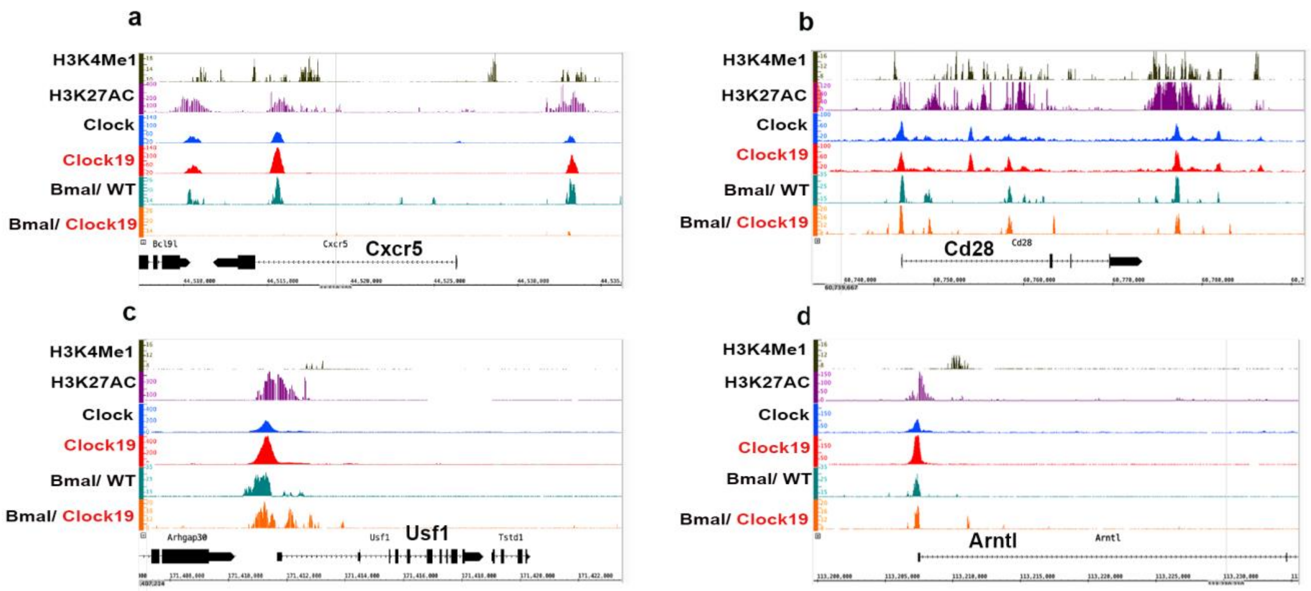

Supplementary Figure 3. CLOCK and BMAL1 chromatin occupancy at representative circadian and immune gene loci.

Genome browser tracks showing ChIP–seq profiles for H3K4me1, H3K27ac, CLOCK, CLOCK$^{\Delta 19}$, BMAL1 in wild-type cells, and BMAL1 in *ClockΔ19* cells at representative loci.

(a) Cxcr5 locus illustrating enhancer-associated CLOCK–BMAL1 binding coinciding with regions marked by H3K4me1 and H3K27ac.
(b) Cd28 locus displaying CLOCK and BMAL1 occupancy within enhancer regions associated with immune activation pathways.
(c) Usf1 locus showing promoter-proximal CLOCK and BMAL1 binding at a transcription factor involved in circadian and transcriptional regulation.
(d) Arntl (Bmal1) locus illustrating promoter-associated binding and chromatin marks characteristic of circadian transcriptional regulators.

Gene models are shown at the bottom of each panel. Signal tracks represent normalized ChIP–seq read density across the indicated genomic regions.

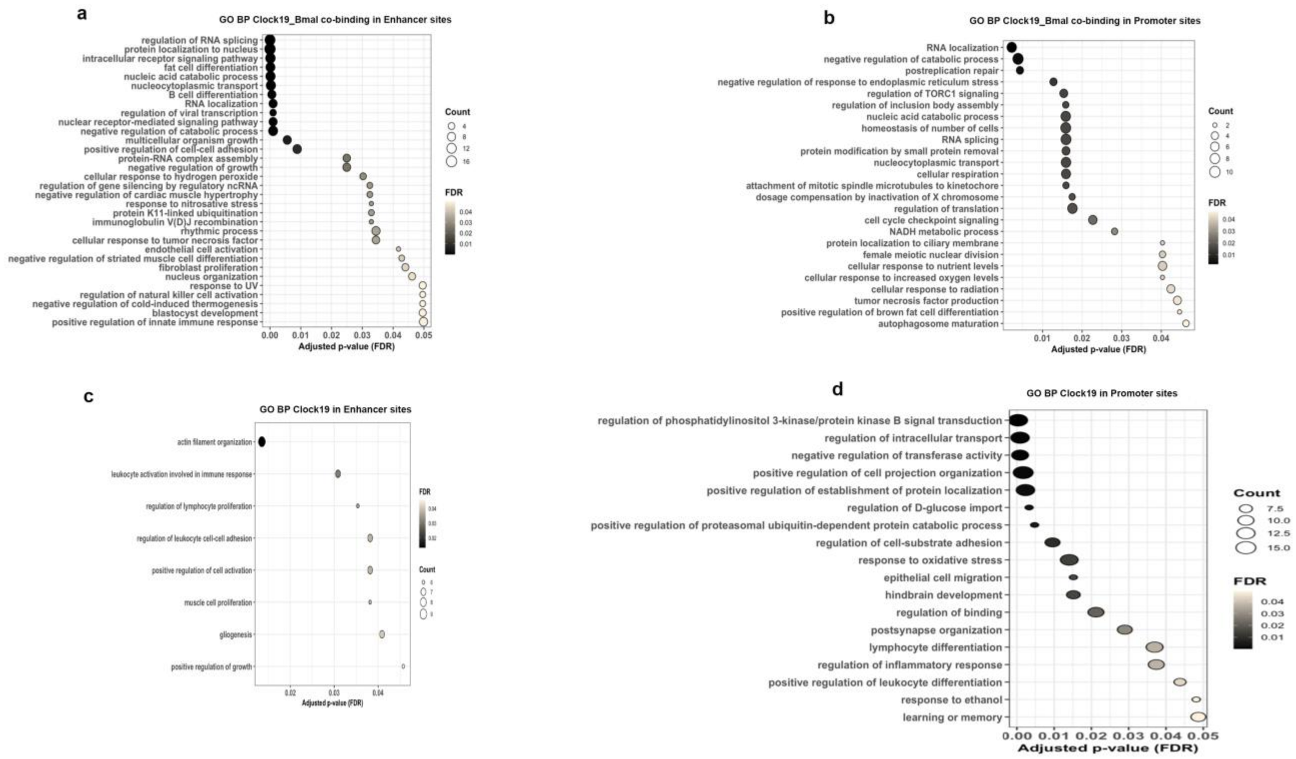

Supplementary Figure 4. **Gene ontology analysis of CLOCK and BMAL1 chromatin binding sites.**

Gene Ontology (GO) biological process enrichment analysis of genes associated with CLOCKΔ19 and BMAL1 chromatin occupancy at enhancer and promoter regions.

(a) GO enrichment of genes associated with CLOCKΔ19–BMAL1 co-binding at enhancer regions**,** highlighting pathways related to RNA processing, intracellular signaling, immune regulation, and cellular stress responses.

(b) GO enrichment of genes associated with CLOCKΔ19–BMAL1 co-binding at promoter regions**,** revealing enrichment for pathways involved in RNA localization, post-replication repair, nucleic acid metabolic processes, and transcriptional regulation.

(c) GO enrichment of genes associated with CLOCKΔ19-specific binding at enhancer regions**,** indicating immune-related processes including leukocyte activation, lymphocyte proliferation, and inflammatory signaling pathways.

(d) GO enrichment of genes associated with CLOCKΔ19 binding at promoter regions**,** highlighting pathways involved in intracellular signaling, protein catabolism, immune cell differentiation, and inflammatory responses.

Dot size represents the number of genes (Count) associated with each GO term, and color intensity indicates the FDR**.**

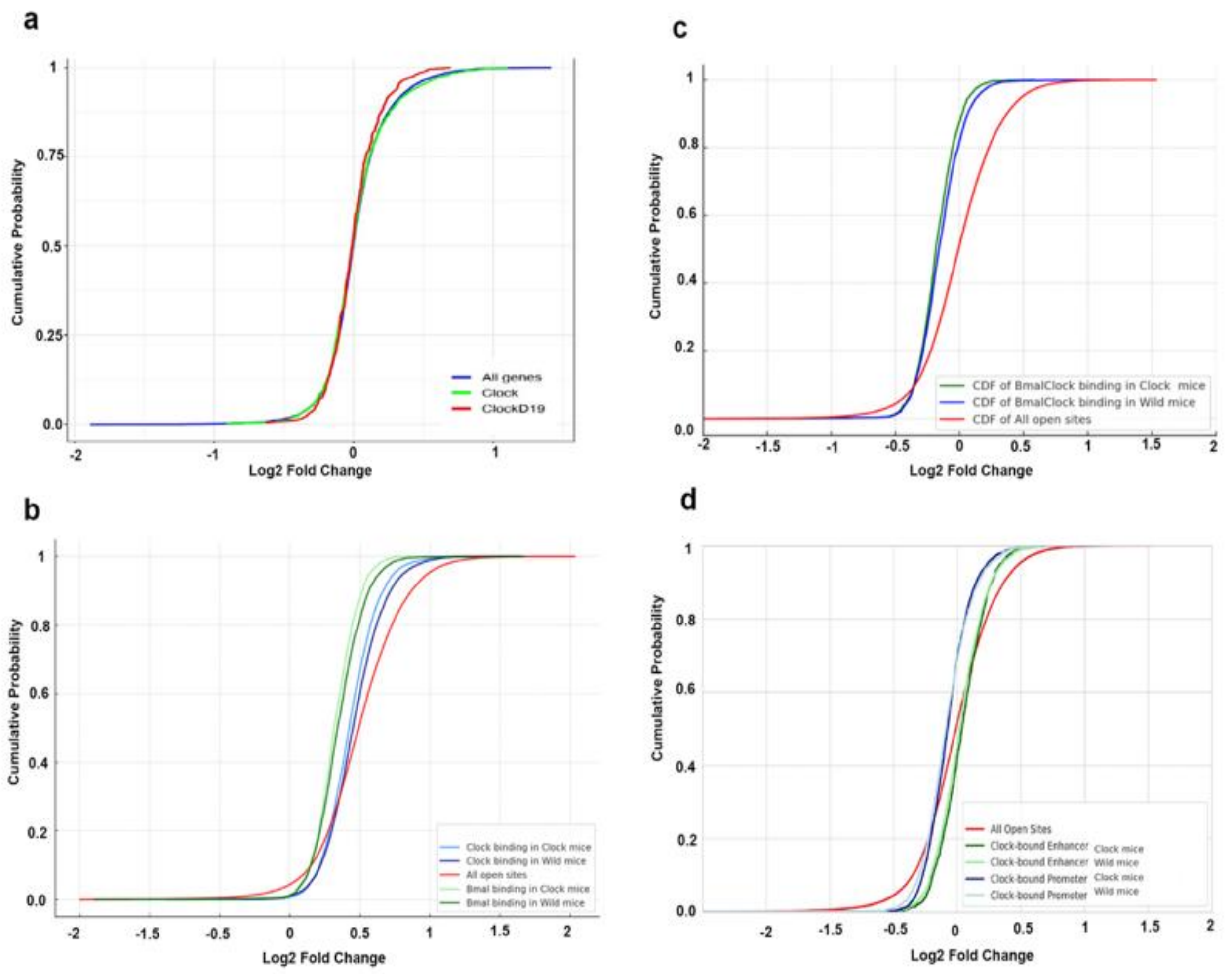

Supplimentary Figure 5- Integration of data: a) Cumulative distribution function (CDF) plot of log$_2$ fold changes in expression for genes bound exclusively by CLOCK or CLOCK$^{\Delta19}$.

b) Reduced global chromatin accessibility at regions exclusively bound by CLOCK or BMAL1 in WT and Clock$^{\Delta19}$ cells, usingCumulative distribution plot (CDF) of log$_2$ fold changes, (c) and bound by CLOCK-BMAL1 hetrodimer in WT and Clock$^{\Delta19}$ cells. (d) Clock or Clock$^{\Delta19}$ reduce promoter accessibility more than enhancer accessibility; CDFs of log$_2$ fold changes in chromatin accessibility at promoter and enhancer regions exclusively bound by CLOCK or CLOCK$^{\Delta19}$ in WT versus Clock$^{\Delta19}$ cells.
